# Outcomes of thyroidectomy for secondary thyroid malignancies, a single center experience

**DOI:** 10.1007/s12672-024-00967-5

**Published:** 2024-04-04

**Authors:** Yunushan Furkan Aydoğdu, Emre Gülçek, Çağrı Büyükkasap, Hasan Bostancı

**Affiliations:** 1 Department of General Surgery, Bandırma Research and Training Hospital, Balıkesir, Turkey; 2 Department of General Surgery, Polatlı Duatepe State Hospital, Ankara, Turkey; 3 Department of General Surgery, Gazi University Faculty of Medicine, Ankara, Turkey

**Keywords:** Renal cell carcinoma, Squamous cell carcinoma, Metastasis, Thyroid

## Abstract

**Background:**

Metastasis of primary malignancies of other organs to the thyroid gland is a rare condition that may pose a diagnostic challenge. In this study, we aimed to report the clinicopathologic features and outcomes of patients treated for secondary thyroid malignancies in our center.

**Materials and methods:**

The results of patients who underwent thyroidectomy in our clinic between 2015 and 2023 were evaluated retrospectively. Four patients who met the inclusion criteria were evaluated.

**Results:**

The primary tumor was renal cell carcinoma in 2 patients and squamous cell carcinoma of the lung in 2 patients. Median age was 64 years (min:59–max:69). The median nodule diameter was 2.85 cm. Extrathyroidal spread was present in 1 patient with renal cell carcinoma metastasis. The other patient with renal cell carcinoma metastasis had lymphatic invasion. Vascular invasion was detected in 1 patient with renal cell carcinoma metastasis and 1 patient with squamous cell carcinoma of the lung metastasis. Surgical margin positivity was present in 1 patient in each group. The median overall survival time was 27 (min:10–max:44) months in the renal cell carcinoma group and 11 (min:6–max:16) months in the squamous cell carcinoma group. After the diagnosis of primary renal cell carcinoma, one patient metastasized to thyroid tissue 43.00 and one patient 94.00 months later. In the squamous cell carcinoma group, one patient showed metastasis to thyroid tissue 6.00 months after the primary diagnosis, while the other patient was first diagnosed with metastatic tissue.

**Conclusions:**

Metastasis to the thyroid gland is a rare phenomenon with an incidence of 0.22% in all thyroid malignancies. It may occur before the detection of the primary tumor or during the follow-up of the primary malignancy. Although the overall prognosis is poor, it can be treated surgically.

## Introduction

In the evaluation of patients with thyroid nodules, thyroid examination with palpation, which represents the main step of physical examination, is performed first. In the evaluation of patient history and palpation, malignancy risk factors should be asked and evaluated in order to exclude malignancy. Radiation exposure, family history, etc. are the main questions to be asked during patient history leading to an increase in thyroid malignancies [1]. If a thyroid nodule is palpable on palpation, the hardness of the nodule, its fixation and the presence of a palpable cervical lymph node should be considered [2].

Despite being richly supplied by numerous arteries, other organ metastases to thyroid tissue are very rare [3]. Metastases from malignancies of other organs represent 0.36% of thyroid malignancies [4].

Most studies on this subject to date have utilized autopsy data and case series with surgical follow-up are rare [5, 6]. Therefore, there is a limited number of studies on thyroid metastases from other organ malignancies.

Secondary organ cancers that metastasize to the thyroid gland include kidney, colorectal, lung and breast cancers [7]. It has been demonstrated that approximately 35–80% of this group of patients have multiple organ metastases leading to worsening of the disease course [8, 9].

In recent years, with the advancement of diagnostic and screening methods, the number of cancers that choose thyroid tissue as a secondary cancer has been increasing incidenceally [10, 11]. Unlike primary thyroid malignancies, carcinoma metastases from organs secondary to thyroid tissue do not respond to radioactive iodine treatment. Therefore, surgery is the primary treatment option in these patients [12].

We aimed to evaluate the recent secondary cancers that were diagnosed and thyroidectomized in our clinic because we think that they have not been sufficiently evaluated in the literature and the number of patients has increased in recent years.

## Materials and methods

**Fig. 1:**
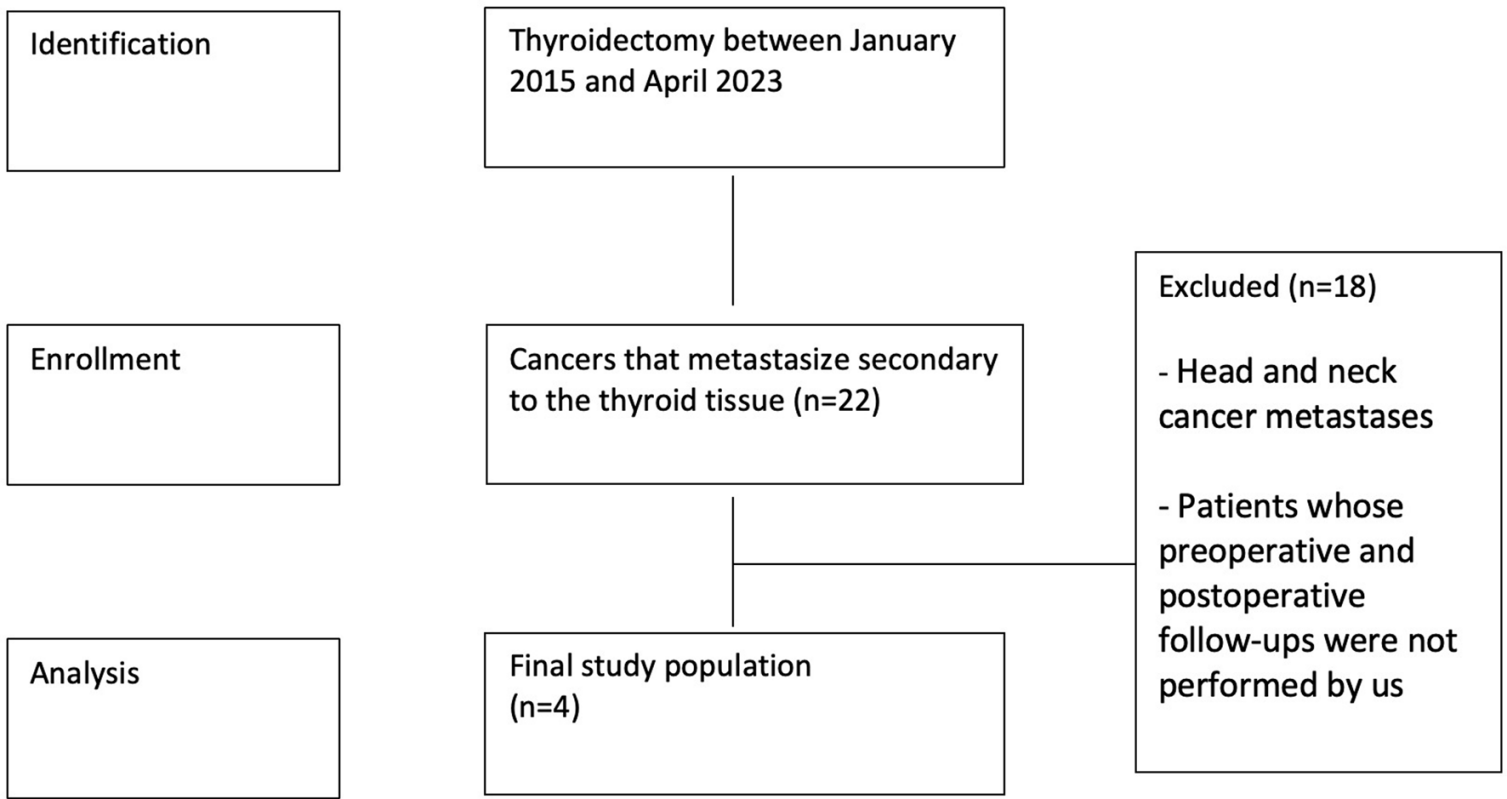
The sample collection scheme

In our study, 1881 patients who underwent thyroidectomy in Gazi University Faculty of Medicine, Department of General Surgery between January 2015 and April 2023 were evaluated. This study was planned as a retrospective, single center experience study. Patients in whom other organ malignancies metastasized to thyroid tissue (secondary thyroid malignancies) were evaluated. Metastases of the primary tumoral tissue from anatomically close neighboring organs (head and neck region cancers) that may occur through adjacent organ metastasis (invasion) were excluded from the study. Patients whose preoperative and postoperative follow-ups were not performed in our hospital were excluded from the study (Fig. 1). The included Patients were evaluated. In 2 patients the primary malignancy was renal cell carcinoma and in 2 patients squamous cell carcinoma of the lung. In 2 patients with renal cell carcinoma, metastatic status was detected during follow-up (thyroid ultrasonography). Thyroid fine needle aspiration biopsy was performed from the relevant nodule. And total thyroidectomy was performed. Patients with squamous cell carcinoma metastasis were evaluated. In one patient, metastasis was diagnosed as a result of thyroid fine needle aspiration biopsy performed during cancer follow-up. Total thyroidectomy was performed. In the other patient (surgical indication: multinodular goiter), the initial diagnosis was made by pathologic examination of the specimen after total thyroidectomy. All 4 patients in the study had metastases only in the thyroid tissue (isolated thyroid metastases).

The demographic characteristics of the patients, the location of the primary tumor, the time elapsed after diagnosis until the occurrence of thyroid metastasis, the disease-free survival time, and the characteristics of the secondary tumoral tissue were evaluated descriptively.

Approval for this study was obtained from the Institutional Review Board Ethics Committee. This study was conducted in accordance with the Declaration of Helsinki.

### Statistical analysis

Statistical analyses were performed using Statistical Package for the Social Sciences (SPSS) (version 26.0, SPSS Inc, Chicago, IL, USA) program. Descriptive statistics of the numerical data obtained in the study were analyzed and numerical parameters were expressed as median (min–max).

## Results

During the study period, 1881 patients who underwent thyroid surgery in our clinic were retrospectively evaluated. There were 22 patients who underwent thyroidectomy for metastatic cancers. Among this patient population, 16 (72%) patients were excluded because of metastatic head and neck cancer. 2 (9%) patients were excluded from the study because their postoperative follow-up was not performed in our hospital. The patient population included in our study included a total of 4 patients, 2 males (50%) and 2 females (50%). The median age of the patient population was 64 years (min:59–max:69).

Among the patient population, the primary malignancy was renal cell carcinoma in 2 patients and squamous cell carcinoma of the lung in 2 patients.

When the metastatic nodular lesions in metastatic thyroid tissue were evaluated within themselves, the median nodule diameter was 2.85 cm.

Extrathyroidal spread was present in only 1 (25%) patient and this patient was in the renal cell carcinoma metastasis group. Lymphatic invasion was seen in only 1 patient (25%) and this patient was in the renal cell carcinoma group. When the vascular invasion status was evaluated, it was seen that invasion was present in 2 patients. And 1 of these patients was in the renal cell carcinoma group and 1 was in the squamous cell carcinoma group. Surgical margin positivity was present in 1 patient in each group.

The median overall survival time in the renal cell carcinoma group was 27 (min:10–max:44) months.

In the squamous cell carcinoma group, the median overall survival time was 11 (min:6–max:16) months. No mortality was detected in any patient during the evaluation. No other organ metastasis occurred in any patient after surgery for thyroid organ metastasis. No recurrence of thyroid tissue was observed in any patient during follow-up.

One patient metastasized to thyroid tissue 43.00 and one patient 94.00 months after the primary diagnosis of renal cell carcinoma. In the squamous cell carcinoma group, one patient showed metastasis to thyroid tissue 6.00 months after the primary diagnosis, while the other patient was first diagnosed with metastatic tissue (Table 1).

**Table 1:** Clinicopathological characteristics of patients who underwent thyroidectomy for secondary thyroid malignancies

Patient number	Patient 1	Patient 2	Patient 3	Patient 4
Age (year)	64.00	69.00	64.00	59.00
Sex	Female	Male	Female	Male
Primary malignencies	Renal cell carcinoma	Lung squamous cell carcinoma	Renal cell carcinoma	Lung squamous cell carcinoma
Metastatic nodule diameter (cm)	4.00	9.00	1.70	0.50
Extrathyroidal spread	Positive	Negative	Negative	Negative
Lymphatic invasion	Negative	Negative	Positive	Negative
Vascular invasion	Negative	Positive	Positive	Negative
Surgical margin	Positive	Negative	Positive	Negative
Time to metastasis after diagnosis of primary malignancy (month)	94.00	0.00	43.00	6.00
Overall survival after diagnosis of metastases (month)	44.00	16.00	10.00	6.00

cm: centimeter

## Discussion

Thyroid tissue metastases from primary carcinomas of other organs represented a very small population among all thyroid surgeries. Balta et al. [13], this rate was 0.69% and Calzolari et al. [14] contributed to the literature by finding 0.15%. In all of our thyroidectomies, we found that metastatic carcinomas of other organs represented a population of 0.31%, which is consistent with the literature.

Tang et al. [7] reported an approximately sixfold difference between the incidence of thyroid metastases of lung carcinoma and renal cell carcinoma, while Nixon et al. [12] and we found the incidence to be equal in our study.

Nixon et al. [12], patients with thyroid metastases have an asymptomatic course. We did not detect any significant symptomatic findings before surgery.

Ghossein et al. [4] found a median age of 63.00 years in their population. In our study, the median age was found to be 64.00 years. Similar to this study, the female/male ratio was found to be 1 in our study.

When we searched the literature, we could not find a study on nodules with malignant cells in thyroid tissue in other organ metastasis to the thyroid gland. According to our data, the median diameter was 2.85 ± 3.75 cm. Likewise, when we evaluated the extrathyroidal invasion, lymphatic invasion and vascular invasion status of the patients included in our study population, we found extrathyroidal invasion with a frequency of 25%, lymphatic invasion with a frequency of 50% and vascular invasion with a frequency of 50%. However, there was no relevant study in the literature. We think that this limitation is due to the fact that studies are generally performed on autopsy series rather than clinically [5, 6] and the working patient group is very rare [3, 13, 14].

The overall postoperative survival of carcinoma metastases from organs secondary to thyroid tissue has been reported to be approximately 24 months [12, 15]. In our study, the median overall survival was 13 months. The limitation of the number of patients in all studies prevents a clear median value from being given.

Ljungberg et al. [16] recommend metastasectomy for renal cell carcinoma metastases except for metastatic areas treated with radiotherapy. Squamous cell carcinoma metastases, on the other hand, have poor overall survival [17]. We observed that disease-free survival after surgery was higher in the renal cell carcinoma group than in the squamous cell carcinoma group, but the time from primary diagnosis to metastasis was also longer. In our study, no patient experienced mortality during the follow-up period. Cichoń et al. [3] found no mortality in patients with isolated thyroid metastases. This was supported by our study.

Medas et al. [18] published a case report in which they evaluated isolated thyroid metastasis. In their study, they recommended total thyroidectomy if there is no metastatic condition elsewhere. They even stated that lobectomy is also an option in these patients if appropriate. Congui et al. [19] and Cichoń et al. [3] also recommended total thyroidectomy in patients with isolated thyroid metastases. We performed total thyroidectomy in all of these patients. The prognosis was good after total thyroidectomy, but we could not evaluate the feasibility of lobectomy if appropriate, because total thyroidectomy was indicated in the patients included in the study.

Although thyroid tissue is an organ rich in blood supply, it is rarely the site of metastasis [20].

This situation is surprising. Nevertheless, when malignancy is suspected in thyroid tissue in patients with a history of primary other organ carcinoma, the possibility of a metastatic focus should be considered before considering it as secondary.

In our study, no recurrence and mortality were detected in secondary thyroid malignancies after total thyroidectomy. As a result, we think that total thyroidectomy may be an adequate treatment option for secondary thyroid malignancies.

## Conclusion

Since it is very rare, studies on other organ metastases to the thyroid gland are limited. Although these studies give an idea about the approach, they are considered to be insufficient when evaluated together in general terms. Nevertheless, when malignancy is suspected in thyroid tissue in patients with a history of primary other organ carcinoma, the possibility of a metastatic focus should be considered before considering it as secondary.

Although we, as our clinic, have shared our experiences in the last seven years with the literature, we have not been able to reach a sufficient number of patients to lead the medical approach. In time, the literature will develop with the presentation of review studies of clinical patient studies.

## Data Availability

The database of this study is open to sharing. It can be obtained from the authors upon request.
